# University of London

**Published:** 1831

**Authors:** 


### UNIVERSITY OF LONDON.

STATEMENT OF THE FACTS OF HIS CONNEXION WITH THE UNIVERSITY OF LONDON. By *Granville Sharp Pattison*, Surgeon, &c. &c. &c. late Professor of Anatomy and Surgery in the University. Octavo, pp. 44. London, August, 1831.

*Resolved*,—"That Professor Pattison be, and he is hereby removed, from his situ-"ations of Professor of Anatomy and Surgery in this University.

*Resolved*,—"That in taking this step, the Council feel it due to Professor Patti-"son to state, that nothing which has come to their knowledge with respect to his "conduct has in any way tended to impeach either his general character or profes-"sional skill and knowledge."—*Minute of Council of July* 23, 1831.

THE sun of the LONDON UNIVERSITY rose under circumstances that promised a bright and cheerful, if not a resplendent day. Sanguine hopes were entertained that an institution was formed, untrammelled by monkish ignorance and aristocratic pride, which would diffuse useful information, in accordance with the advanced state of art, science, literature, and civilisation, while it gave full and fair play to talent and to industry. There were, however, discerning eyes, even among its friends and supporters, that saw certain maculæ on the face of this Sun of Knowledge, which portended storms, and clouds, and rain. The determination to dissever religious instruction from literature and science, was, it was said, a hazardous speculation, and an ominous feature in the New Institution. Such a dissociation might have no injurious effects in FRANCE, where the solemn truths as well as the outward forms of Christianity were treated

---

with little respect, or rather with open mockery; but it was doubted whether England was yet so far enlightened by fifteen years friendly intercourse with her neighbours, as to admit this deviation from the established regulations of academic bowers, without repugnance and strong opposition. A very short time shewed that these prophecies were well-founded; for there cannot be now a question, that the above objection has crippled the new institution, and contributed materially to its avowed want of success. Still, with this heavy drawback, the New University might have flourished, had it not exhibited two other important defects—want of wisdom in its management—and want of moral justice, we had almost said, want of common *honesty*, in its decisions. The Council of the University have signed, sealed, and delivered this sentence of condemnation on themselves, in the epigraph which we have placed at the head of this article! In avoiding, with horror, the ancient maxim "fiat justitia, ruat cœlum," they seem not to have dreamt that there was another rock on which the vessel might split—"fiat INJUSTITIA, RUAT UNIVERSITAS." It is very true that PUBLIC BODIES, like nations and states, are far more selfish than individuals. They never do any thing *designedly* against their own interests; whereas individuals, in all ages and countries, have sacrificed self for the good of others. But these public bodies, nations, and states, have very rarely effected, in the end, their own interests by INJUSTICE—by immolating RIGHT at the shrine of EXPEDIENCY. A day of reckoning and of retribution almost always comes, sooner or later—and that day has arrived very quickly with the New University.

We do not believe that there is an instance on record, where dissention, mismanagement, intrigue, and want of faith, so soon and so effectually sapped the foundation and blasted the fame of a great Public Institution, as in the instance now before us. It would seem that DISCORD lit her brightest torch at the very birth of the University, and has kept it fiercely burning ever since. The resignation, in disgust, of so many of her professors and officers, and in such a short space of time, is most unprecedented—and highly disgraceful to the Institution. We shall not, however, notice any other matters or proceedings than those connected with the medical department, and with the transaction now before us. The RIGHTS or the WRONGS of one individual are of just as much consequence as those of five thousand. Any infringements on the *former* or perpetration of the *latter* demand as much investigation, where a single individual is concerned, as where a multitude are the victims. We shall now, therefore, endeavour, in as brief a manner as possible, to put our readers in possession of the leading features of Mr. Pattison's case, in order that they may be enabled to judge for themselves, conceiving it to be of great importance that the medical profession at large should come to some settled opinion respecting the merits of the question at issue.

As Mr. Pattison's statement may be considered as an *exparte* one, it is necessary to premise that he submitted the proof-sheets to one of the Council, and the only medical member of it—consequently one most capable of judging—Dr. Birkbeck. This gentleman has declared, in writing, that Mr. Pattison's statements are "*always substantially and generally minutely correct, and remarkably free from exaggeration.*" A further evidence of their truth has been furnished by the fact that, at the public meeting of the proprietors held on the 20th August, for considering his case, although his "Statement" had been published a week, the Council were unable to invalidate or put aside any of the charges he has advanced against them.

I. Mr. Pattison commenced his anatomical career in Glasgow, at the age of twenty years; and although he had Dr. Jeffery for his rival, yet his class augmented so rapidly that he was frequently obliged to refuse tickets from want of room, even in a large theatre.

II. With the private reasons which induced Mr. P. to give up so good a prospect, we have nothing to do. Suffice it to say that he was invited to Philadelphia, and there, being disappointed of the professorship which he expected, in the face of great difficulties—we may also suppose, *prejudices*, he soon, as a private teacher, attracted a class of more than 190 students, and secured so high a reputation as to induce the Regents of the University of Maryland to send a deputation to request him to accept of a professorship in their Institution. In this University Professor Pattison lectured for five years, and, by his talents as a teacher of anatomy and surgery, encreased the numbers of the medical class from 70 to above 300, the emoluments of which, with his private practice, averaged full 2000 pounds per annum.

III. It was from this situation that he became candidate for the Chair of Anatomy and Surgery in the London University; and was successful, although Mr. Charles Bell, Mr. Mayo, and several others, were competitors for the office. It can hardly be supposed that there was any favouritism exercised in his appointment—indeed there was probably no lack of calumny exercised by his enemies (we do not mean his competitors) on this appointment.

IV. It appears that some discredit has been attempted to be thrown on the *testimonials* produced on this occasion by Mr. Pattison, on the grounds that any blockhead may, by the assiduity of himself and friends, procure testimonials of talent and acquired knowledge to any amount. This is unfair—especially in the present case, where the *facts* of Mr. Pattison's success, as a teacher, were unanswerable documents in his favour. But independent of these testimonials, Mr. P. addressed letters to Lord Brougham and T. Campbell, Esq. earnestly soliciting the trial by *concours*, for the decision of the election. This was declined by the Council, as unnecessary.

V. Immediately on his appointment, he was requested to visit Germany, to collect preparations, &c. with a promise of a salary of £300 per annum, till the University should open. He spent £130. in his travels—and by a quibble, he was only rewarded with £100. at the end of a year and a half, employed on University business! The *punica fides* was thus early evinced by the London University!

VI. But the greatest grievance, of which Mr. P. has to complain, and which indeed led to all the subsequent evils of the case, the appointment of a *demonstrator* of anatomy quite independent of—and, in fact, in opposition to, or, at least, in rivalry of—the lecturer on the same subject. Whoever has spent even a week in a dissecting-room must have observed the advantages which a demonstrator has over the lecturer, for various and obvious reasons. The moment the unity of interest and reputation between these two personages is destroyed, from that moment ruin is threatened to one or both of them. Unfortunately Mr. Pattison and Mr. Bennett were placed in this false position, and the consequences have been fatal to one at least of them, while the conflicting passions of a mind ill at ease contributed no doubt to hasten the end of the other. If the evil genius of the University had been given a carte blanche for sowing the seeds of discord and destruction in the anatomical department of the Institution, he could not have devised a more certain method of effecting his purpose than by the arrangement which the Council ordained between Messrs. Pattison and Bennett. Dr. Birkbeck, the only medical member of the Council, and whose opinions ought to have had weight, was loud in his reprobation of this measure. With something like the spirit of prophecy, he predicted most of the heart-burnings and consequences which ensued.

VII. Among the early demonstrations of hostility to Mr. Pattison, were the following. Although the lectures went on prosperously for the first few months, yet *anonymous* writers began to make their way to the Council—were entertained—the complaints investigated—and the charges found groundless. Again the system of anonymous accusations commenced—again the ear of Dionysius was opened in the star-chamber of the London University—again the secret conclave of the Inquisition met, and perused the anonymous slanders—and again an investigation was set on foot! The subject was referred to Mr. Joseph Hume, who declared that " there was no legitimate ground of complaint, either as related to the supply of subjects, or as to the mode in which Mr. Pattison conducted his lectures."

The first session having come to a close, at the distribution of honours, Mr. P. had occasion to read a passage from one of the answers of the gentleman who obtained the gold metal, and to commend it warmly.

"This was, however, displeasing to Mr. Bennett; and as Dr. Turner, who sat next to him on this occasion, observes, '*His conduct was exceedingly indecorous. His expressions of disapprobation were so loud, and his gestures so eager,*' that Dr. Turner '*was quite alarmed, and felt it necessary not to lose a moment in pacifying him.*' The intention of this manifestation of disapprobation was easily interpreted. It was intended to convey the impression to those around him, that what I read was in error, and that the Professor of Anatomy was so ignorant of his subject as to be unable to detect the anatomical blunders it contained. The matter was not permitted to rest here: my enemies, flattering themselves that by giving a sufficient circulation to this story, which they considered an incontestible evidence of my ignorance of anatomy, my reputation as an anatomical teacher would be ruined, took great pains to give it ample circulation. Who they were, and what measures they pursued to accomplish their purpose, I shall not stop to state; but their exertions were most successful, and almost every medical man in the kingdom had this pretended evidence of the ignorance of the Professor of Anatomy of the University of London detailed to him!" 5.

An open rupture now, of course, took place between the professor and his demonstrator, and each addressed the Council. In Mr. Bennett's letter, he reiterated the charge of ignorance of anatomy against Mr. Pattison, and the following passage will put our readers in possession of the specific instances adduced by the accuser.

"Paper marked No. 4 contains a literal copy of the passage which I read; and the passages which Mr. Bennett has marked as incorrect are underlined.

1st. My pupil states,—that the fifth pair of nerves are nerves of '*sensation.*' Mr. Bennett asserts that the fact is not so. I must confess I could hardly have expected such an assertion from Mr. Bennet. It is true Mr. Bell, in his first experiment, was deceived, and did publish, that the branches of the fifth nerve bestowed *motion and sensation* on the parts on which they were ramified. This *mistake* was, however, speedily discovered by M. Majendie; Mr. Mayo, and every anatomist and physiologist are now satisfied that the fifth pair of nerves are nerves of sensation. The third branch of the nerve is joined by a motor twig, after it has passed out of the skull; but as the first and second branches are merely nerves of sensation, in speaking *generally* of the nerve as a *whole*, it would be decidedly incorrect to say that it was a nerve of '*sensation and motion.*'

2nd. My pupil has stated, '*the white filaments do not enter into its formation;*' viz. the ganglion of Gasserius. Mr. Bennett asserts, that Mr. Jones is incorrect in the assertion that the whiter filaments do not enter into this ganglion. Bichat, Cloquet, and all the most distinguished anatomical writers, are of the same opinion as my pupil, and of course differ from Mr. Bennett. The following extracts from '*Bichat, Anatomie Descriptive,*' tome iii. p. 164:—'Lorsqu'on renverse de dedans en dehors le faisceau aplati des filets du nerf, et le renflement auquel ils se terminent, on voit, comme l'a fait observer Prochaska, qu'entre eux et le rocher *les filets antérieurs* dont nous avons parlé restent *totalement* distinct. Leur volume, LEUR BLANCHEUR, leur isolement, les font reconnoître, IL NE S'UNISSENT POINT AU RENFLEMENT (ganglion);'—and from Cloquet's '*Anatomie Descriptive,*' tome ii. p. 100:—'LES FILETS PLUS BLANCS, ILS NE S'ENGAGENT PAS DANS LE GANGLION,'—will suffice to prove that the description of my pupil is perfectly correct.

3rd. The last passage in the description, marked by Mr. Bennett as an error, is the following line: 'It *then* (the ophthalmic branch) passes through the sphenoidal fissure, and divides into three branches.' Mr. Bennett says it divides before it passes into this fissure. Now both descriptions may be said to be correct. One anatomist may say, that the nerve has not passed through the fissure, until it has perforated the dura mater; whilst another may assert, that as the dura mater is reflected through the sphenoidal fissure, and as the nerve does not divide until it touches that membrane, it has, in truth, passed through the fissure before the division takes place. In Mr. W. Bennett's translation of M. Bayle's 'Manual of Anatomy,' the text-book which Mr. Bennett RECOMMENDS AS A GUIDE to his pupils, the description of the point of division is in strict accordance with that given by my pupil, p. 269, 'receiving a twig from the superior cervical ganglion, and traversing the sphenoidal fissure,—IT AFTERWARDS divides into three branches,' &c." 9.

From the above, it is but too evident that the late Demonstrator was actuated by some hostile spirit against Mr. Pattison—indeed we have heard him, even on his death-bed, use such bitter expressions towards his colleague, that we have more than once or twice remonstrated with him on his hostility to a fellow-labourer in the same Institution. With the reasons or motives which urged the late Mr. Bennett to this conduct, we have no knowledge. They lie concealed with him in the lonely grave!

VIII. Mr. Horner, the warden of the University, now comes on the arena. His invitation to Mr. Charles Bell to take Mr. Pattison's place is a curious transaction already known to the public. The third session was marked by an increase of annoyance to the unfortunate Professor of Anatomy. He was assailed by open and concealed accusers, and at this time four of his colleagues, Drs. Conolly, Thomson, Davis, and Turner, formed themselves into a committee and investigated the charges against Mr. P. They examined all the more intelligent and diligent students, and the result was a complete conviction that the charges were utterly groundless. They conclude by affirming "that this enquiry has furnished strong matter of suspicion that the charges have originated in feelings which are too personal in their nature to deserve the countenance of the council."

From this time the train of transactions is quite sickening to think of—and we cannot bear to give their details. A Mr. Eisdel, whose examination, it is said, evinced the most astounding ignorance, addressed a letter to the Council, declaring it as *his opinion* that Mr. Pattison was incompetent to teach anatomy. The Council kindly considered this student's letter, and as kindly wrote to him that they could not institute an examination into the charges upon the representation of "*one* pupil"—which was as much as to say, you must get half a dozen pupils to join you, and *then* we are at your commands!! The invitation was readily caught at, and, accordingly about fourteen or fifteen pupils joined with Eisdell and Dr. Thomson, jun. in a coalition against the devoted Professor of Anatomy. Before another investigation could take place the students had dispersed into the country, but not before they were invited to send their charges *in writing* to the Council. This was fortunate. They could not combine under an artful leader, and their accusations were all contradictory! One accused him of not lecturing on a particular organ—another of lecturing too much on that same organ! One affirms that he was not minute enough on the bones—another that he spent a great deal too much of his time on osteology! In short, their accusations were nothing but a tissue of contradictions which would have turned them out of any court of justice in Christendom. This was "too bad." The Council were almost ashamed of themselves, and *compelled* to come to a decision that the charges against Professor Pattison were "utterly groundless."

IX. The Council being at last, when too late, convinced of the impolicy of making the Demonstrator of Anatomy independent of the Professor, applied to Mr. Pattison for advice. He offered to give Mr. Bennett (spontaneously) one half of his professorship, provided that, in all announcements, &c. it should be distinctly stated as a voluntary surrender of the same, and not as forced upon him by the Council. Here again the punica fides became manifest. They faithfully promised the foregoing stipulations—but forgot to perform them! They did not indeed state that Mr. P. was forced to this measure, but they omitted all mention of the voluntary nature of the gift. Like Jupiter of old, they—

Heard the prayer,
Accorded half, while half was lost in air.

Shortly after the investigation above alluded to, Mr. Pattison was appointed to the chair of surgery, shewing pretty plainly that no spot of blame attached to his character. As Dr. Thomson, junr. had taken an active part in the insurgency of the pupils, he was prohibited from entering the University; but Mr. Horner, on his return from Scotland, quickly annulled the order, and on his own responsibility, in direct violation of the order of Council, admitted Dr. T. who made good use of his time in setting the students again to work at the old trade. Mr. Horner, in the plenitude of his power, little thought that his turn was coming, and that, ere long, his own wings would be clipped in the London University! Will it be believed, that Mr. Horner, the Warden, wrote or dictated a letter for the insurrectionary students to forward to the Council!! The scenes of riot, insubordination, and misrule on the part of the students—the incapacity of the Council—and the duplicity or worse of the Warden, baffle description, and would disgrace the pages even of a common newspaper. We have not room,—and indeed we have not patience to condense the shameful series of outrages on decency, justice, discipline, and common sense, which are here displayed. Our blood boils when we think of them; but they will bring on the heads of their authors a condign punishment, the utter ruin of the school—at least of the medical *school*—for the name of UNIVERSITY it does not deserve.

X. Harassed with the insubordination of the pupils and the inquisitorial conduct of the Council, Mr. Pattison proposed that a short-hand writer should take down his lectures verbatim, and thus afford the University and the public an opportunity of judging of their nature. The Council refused, on the ground that —"*they had no doubt as to his ability and fitness for the discharge of the duties of his professorship.*" But *after* this decision, a kind of secret committee—an imperium in imperio—sat for several weeks, examining into the question of Mr. Pattison's competency! The climax of ignorance, injustice, and absurdity, is now at hand.

"The injustice of the acts of this Committee is only exceeded by their absurdity. Without taking the pains to ascertain whether I had really committed the errors with which I was charged, they proceeded to investigate whether the charges made did or did not contain anatomical blunders.

Lord King, Mr. William Marshall, and Mr. Merrivale, not one of whom knew a nerve from an artery, constituted themselves the judges of my anatomical pretensions! The proceedings of this Committee became too ridiculous for even the students to stand it. The anatomical engravings belonging to the medical library were carried into the Council Room; and with these before them, and with the assistance of anatomical dictionaries to explain technical terms, these gentlemen gravely deliberated on the amount and correctness of the anatomical knowledge possessed by the Professor of Anatomy!

I have never been able to learn precisely what was the result of the deliberations of this committee. I believe they could not make out a single charge, and getting tired, in about three weeks, of the study of anatomy, they terminated their labours." 28.

It might have been expected, or at least hoped, that the termination of the session would have put an end to the Professor's vexations. But not so. In May of the present year Mr. Pattison received a notice from the Warden that a Mr. Thomas Wilson was about to move the Council that, "it be recommended to Professor Pattison to retire, &c." To this Mr. P. naturally enough demurred, and addressed the Council against the proposed motion. Mr. Wilson did not attend on the day appointed; but a smuggling procedure afterwards took place, and a session of Council was summoned for the purpose of removing Mr. P. from his professorships. This being known to the Professors of the University, a most spirited and energetic remonstrance, from six of the most distinguished Professors, was forwarded to the Council. We wish we had room for this admirable document. One sentence we shall copy—since it ought to be written in letters of gold. "We beseech the Council to bear in mind, *that injustice can never be defended by expediency;* that whatever be the *immediate* effects of the proposed measure, it must, in the long run, be followed by evil, which never fails to attend a departure from strict equity, &c." This protest seems to have staggered the Council. At the meeting, an adjournment *for a month* was determined on, Mr. Pattison's friends clearly understanding that this was only another name for an adjournment *sine die.* At the end of the month (during which a threat and an offer of £200. per annum for five years, were held out as an inducement to voluntary resignation) the trial again came on, and again it failed, as a quorum of the Council could not be collected to perform the dirty and infamous execution! It was now supposed that the Professor would have some respite from persecution and tyranny. But no. The SELECT COMMITTEE (packed friends it would appear of Mr. Horner) went to work, and soon passed a resolution to recommend to the Council that Mr. Pattison should be dismissed! It is hardly necessary to state what has been made so generally known to the public, that this recommendation was adopted and acted on, the Council stultifying and damning themselves for ever by the resolution quoted at the head of this article. It is true that Captain Gowan, one of the Select Committee, protested against the proceedings;—and that a meeting of the proprietors was again called, on the 20th August to reconsider the transaction. This meeting confirmed the tyrannical proceedings of the Council—and thus terminated the first act of the University drama!

Such a result was to be expected from an institution which repudiates religion—violates justice—and openly sacrifices morality to expediency! But injustice is always short-sighted—and truly have the six Professors said that "whatever be the *immediate* effects of this measure, it must, in the long run, be followed by evil, which never fails to attend a departure from strict equity." Yes; the evil day will come—and too soon for the mercenary proprietary, who will find that, however prone to wickedness parents may be, they do not generally choose to educate their sons in dishonorable principles. What parent, we ask, will send his child to a UNIVERSITY where there is no discipline, no religion, no sense of honor or justice—and where a premium is held out for insubordination, faction, misrule, and contempt of all decorum? The fact is, the LONDON UNIVERSITY has sealed its own fiat in its conduct on this occasion. The Professors are treated like servants—and their places are made to be dependent on the judgment or caprice of ignorant students. Well might the six Professors say, in their address to the Council, "that the honour, the respectability, and the rank in society of each of them was involved in the decision of the Council upon this point." Lastly, it is melancholy to think, that a number of youths, whose minds are naturally ingenuous, and imbued with a love of justice as well as generosity, should have been so poisoned by faction from below, and so nurtured into insurrection by imbecility from above, that *they* have played their part, and no unimportant one, in the tyrannical, unjust, and disgusting transaction which we have here put on record. We deem it not necessary to apologise to our readers for the length to which this article has extended. The proceedings of the Council, the Secret Committee, and the Proprietary of the LONDON UNIVERSITY should be extensively known, in order that parents and guardians may be aware of the kind of scholastic discipline, moral precepts, and religious feelings which are likely to be instilled into the minds of youth in that INSTITUTION.

P.S. A second meeting of the Proprietors took place on the 3d of September, when a majority, as might be expected, confirmed the proceedings of the Council. But more than a third of this meeting were in favour of the Professor. Would any man be condemned, if FIVE out of the 12 composing a jury decided in favour of the accused? Certainly not. The Council and their friends were unable to invalidate any part of Mr. P.'s statement.

